# Hybrid Immunity to SARS-CoV-2 from Infection and Vaccination—Evidence Synthesis and Implications for New COVID-19 Vaccines

**DOI:** 10.3390/biomedicines11020370

**Published:** 2023-01-27

**Authors:** Julia R. Spinardi, Amit Srivastava

**Affiliations:** 1 Vaccine Medical Affairs—Emerging Markets, Pfizer Inc., Sao Paulo 04717-904, Brazil; 2 Orbital Therapeutics, Cambridge, MA 02139, USA

**Keywords:** hybrid immunity, SARS-CoV-2, COVID-19, vaccination, infection, mRNA vaccine, booster, reinfection, transmission, breakthrough infection

## Abstract

COVID-19 has taken a severe toll on the global population through infections, hospitalizations, and deaths. Elucidating SARS-CoV-2 infection-derived immunity has led to the development of multiple effective COVID-19 vaccines and their implementation into mass-vaccination programs worldwide. After ~3 years, a substantial proportion of the human population possesses immunity from infection and/or vaccination. With waning immune protection over time against emerging SARS-CoV-2 variants, it is essential to understand the duration of protection, breadth of coverage, and effects on reinfection. This targeted review summarizes available research literature on SARS-CoV-2 infection-derived, vaccination-elicited, and hybrid immunity. Infection-derived immunity has shown 93–100% protection against severe COVID-19 outcomes for up to 8 months, but reinfection is observed with some virus variants. Vaccination elicits high levels of neutralizing antibodies and a breadth of CD4+ and CD8+ T-cell responses. Hybrid immunity enables strong, broad responses, with high-quality memory B cells generated at 5- to 10-fold higher levels, versus infection or vaccination alone and protection against symptomatic disease lasting for 6–8 months. SARS-CoV-2 evolution into more transmissible and immunologically divergent variants has necessitated the updating of COVID-19 vaccines. To ensure continued protection against SARS-CoV-2 variants, regulators and vaccine technical committees recommend variant-specific or bivalent vaccines.

## 1. Introduction

Given the current state of the COVID-19 pandemic after 2+ years, a substantial proportion of the global population has been infected with SARS-CoV-2, and a comparatively smaller, but growing, proportion has been vaccinated [1]. SARS-CoV-2 infection-induced immunity was demonstrated early during the pandemic by analyzing sera and T cells derived from convalescent individuals, COVID-19 decedents, and challenge experiments in non-human primates [2,3], all of which subsequently became the foundation for vaccine development efforts. Immunity obtained from infection continues to be characterized both by conducting large seroprevalence studies, and by measuring immunogenicity in smaller specific cohorts. COVID-19 vaccines demonstrated high efficacy in randomized clinical trials [2], which translated into high real-world vaccine effectiveness (VE) in the short term, as part of mass vaccination programs globally. In the long term, effectiveness of primary vaccination wanes with respect to protection against infection and symptomatic disease, but more durable protection against severe disease has been consistently observed. A series of emerging SARS-CoV-2 variants transformed the COVID-19 pandemic with differing phenotypes in terms of transmissibility, virulence, and antigenicity—e.g., higher transmissibility with Delta (B.1.617.2), and increasing antigenic divergence with Beta (B.1.351) and Omicron (B.1.1.529) variants [3]. Additional booster doses in homologous or heterologous schedules have restored waning VE post two-dose primary vaccination, and have been instrumental in enabling the breadth of protection against the Omicron variant [3,4], which has emerged as the most transmissible and antigenically divergent variant to date.

**Figure 1:**
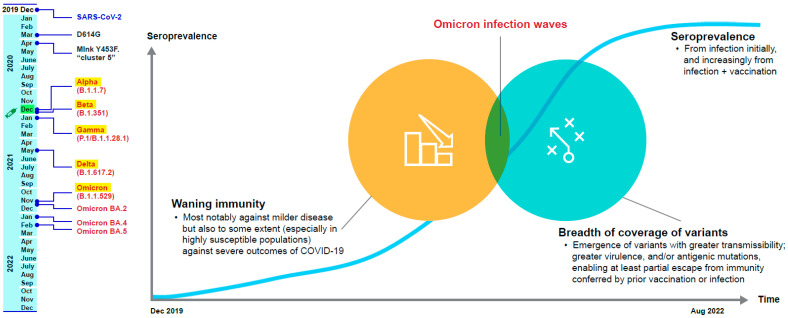
The challenges of maintaining protection against SARS-CoV-2 [10,11,12,13,14,15,16,17,18].

The authorized or approved COVID-19 vaccines are based on the original (or ancestral) Wuhan SARS-CoV-2 strain and its spike protein. However, the recent and current circulating SARS-CoV-2 variants harbor mutations, notably in the spike protein, that confer at least partial antigenic escape from vaccine-elicited immunity [5,6]. At the same time, despite waning immunity, vaccines have retained varied levels of effectiveness against SARS-CoV-2 variants that have emerged to date, with higher-level effectiveness preserved against severe outcomes (hospitalization and death) of COVID-19 than against mild symptomatic disease [7,8,9]. Therefore, the challenge of securing protection against this novel pandemic pathogen—SARS-CoV-2—is to strike a balance between immunity from infection and vaccination that provides a sustainable, broad, protective coverage across variants (Figure 1).

This targeted review of rapidly emerging data summarizes the latest available evidence on SARS-CoV-2 infection-induced and hybrid immunity (infection + vaccination); vaccine-induced immunity has been covered extensively elsewhere and is not included here [4,19,20,21]. We discuss accumulating evidence on the duration of protection conferred by hybrid immunity and cross-protection across variants, and further discuss design, as well as regulatory and implementation considerations for potential in updating COVID-19 vaccine boosters, based on this accrued evidence.

## 2. Search Methods

For the evidence summary, a targeted search was conducted of PubMed, preprint servers (*medRxiv* and SSRN), hand search of selected journals that continue to publish a high volume of COVID-19 immunity research (e.g., *NEJM, JAMA, Nature* family, *Lancet* family, *Science* family), and authors’ collections that identify studies on hybrid immunity to SARS-CoV-2 vaccines, published up to 5 August 2022. The English language articles identified through a search of terms (a wildcard symbol: * was used to broaden the search by finding words that start with the same letters), including *SARS-CoV-2, Sero-epidemiologic Studies, serosurveys, mRNA Vaccines, *Hybrid immunity, *Natural immunity, *Reinfections, *Vaccination, *Vaccines, cross protection, Omicron, naturally acquired immunity, vaccine-induced immunity, were reviewed. The studies identified were classified by the methods used to evaluate immunity, i.e., sero-surveys, immunogenicity assessments measuring B- and T-cell responses, and clinical and real-world effectiveness studies.

### 2.1. Seroprevalence after the First Two Years of the COVID-19 Pandemic

A systematic review and meta-analysis used data pooled from 803 studies to estimate regional and global seroprevalence of SARS-CoV-2 from primary infection (82% of studies reported a 0% vaccination rate) or vaccination (11% of studies reported vaccination in >10% of the population) [22]. Overall, the results showed an 8.2-fold increase in global seroprevalence from June 2020 to July 2021, with approximately 45.2% (35.2% from infection only) of the population having antibodies against SARS-CoV-2. Increases in seroprevalence were observed, concomitant with the waves of Alpha (B.1.1.7), Beta, and Delta variants. Children (<10 years) were found to be less seropositive than adults (20–29 years) overall, and estimates of seroprevalence were higher for low- and lower-middle-income countries.

Seroprevalence measurements have been shown to vary depending on the levels of pre-Omicron vaccination coverage. South Africa and Chile represent two contrasting examples of varying seroprevalence due to infection and vaccination. A study conducted in South Africa from October to December 2021 (i.e., pre-Omicron), wherein only 19% of participants were vaccinated, showed considerably high seroprevalence overall (73.1%), suggesting that this seroprevalence was mainly due to infection [23]. A subsequent study evaluating seroprevalence among blood donors from all provinces in mid-March 2022, estimated that ~98% of South Africans already had some antibodies for SARS-CoV-2 [24]. However, only 10% of the sampled population presented anti-spike antibodies, but not anti-nucleocapsid antibodies, representing the proportion of individuals who were vaccinated and with no previous SARS-CoV-2 infection; limited variation was observed between provinces. On the other hand, a study conducted in Chile during a similar time period of October to November 2021 (pre-Omicron), showed that 97.3% of the surveyed population had antibodies for SARS-CoV-2, and that was strongly associated with vaccination [25]. Chile had high vaccination rates (i.e., 83.8%, 76.9%, and 29.9% coverage for first dose, primary series, and booster, respectively). Overall, 99.1% of vaccinated individuals and 40.9% of unvaccinated individuals were seropositive, and ~100% seroprevalence was reached in individuals receiving booster doses.

Taken together, these studies indicate that the South African population had acquired primarily infection-induced immunity due to high levels of exposure to the virus and low vaccination coverage. Chile, in contrast, had a greater proportion of vaccination plus infection-induced immunity, and a sizeable population of individuals who had only infection-induced immunity. A US cross-sectional study conducted between July 2020 and December 2021 in ~2.4 million blood donors showed that combined seroprevalence from infection or vaccination reached 94.7% by December 2021 [17].

### 2.2. Infection-Induced Immunity

#### 2.2.1. Immune Persistence and Duration of Protection

Two studies were identified that assessed protection against SARS-CoV-2 over time [26,27]. A large-scale study comparing antibody titer decay following infection or vaccination demonstrated that SARS-CoV-2–infected individuals had differing kinetics for waning of B- and T-cell responses [26]. In this study, antibody titers decreased at a slower rate in unvaccinated, previously infected individuals (*n* = 4361), than in fully-vaccinated individuals (*n* = 2653; two doses of BNT162b2). Whereas vaccinated individuals had higher anti-spike IgG antibody titers than previously infected individuals, titers decreased by 38% per month in vaccinated individuals and approximately 12% per month in unvaccinated, previously infected individuals [26]. In another study, SARS-CoV-2–specific memory T-cell responses were sustained for 10 months after symptom onset in infected individuals (*n* = 101), irrespective of COVID-19 severity (7 patients were asymptomatic during infection, 46 had mild symptoms, 25 had moderate symptoms, 14 had severe symptoms, and 9 had critical disease). Furthermore, polyfunctionality and proliferation capacity of T cells was sustained over time, indicating long-term protective immunity after SARS-CoV-2 infection [27].

An elegant retrospective study using Qatar’s national databases provided real-world evidence of the duration and the breadth of protection attained from SARS-CoV-2 infection [28]. Matched cohorts of unvaccinated individuals with and without primary infection were compared with respect to the incidence of reinfection with either Omicron or any non-Omicron variant, and also with respect to COVID-19 outcome severity, between 28 February 2020 and 5 June 2022. Prior to Omicron emergence, primary infection was 85.5% (95% CI: 84.8–86.2%) effective against reinfection with a pre-Omicron variant, rising to 90.5% (95% CI: 88.4–92.3%) at month 7 post-infection and then waning to ~70% by month 16 post-infection. Non-Omicron primary infection showed a much-reduced effectiveness against Omicron reinfection, 38.1% (95% CI: 36.3–39.8%), and also waned over time post-infection. However, primary infection with either Omicron or any non-Omicron variant showed very high (97.3% [95% CI: 94.9–98.6%]) and sustained effectiveness against reinfection-induced severe, critical, or fatal COVID-19 outcomes. A similar retrospective analysis of a cohort (>2 million) derived from a Swedish national registry, comprising unvaccinated individuals with and without immunity from previous infection, demonstrated that infection-derived immunity conferred a 95% reduction in risk of SARS-CoV-2 reinfection (adjusted hazard ratio, 0.05; 95% CI: 0.05–0.05; *p* < 0.001) over the first 3 months and an 87% reduction in risk of COVID-19 hospitalization (adjusted hazard ratio, 0.13; 95% CI: 0.11–0.16; *p* < 0.001) for up to 20 months of follow-up [29].

#### 2.2.2. Breadth of Coverage against SARS-CoV-2 Variants

Two studies illustrated cross-reactive B-cell responses post-infection [30,31]. The first study examined cross-neutralization activity in convalescent sera from infection with SARS-CoV-2 variants (pre-Omicron) [30]. Sera were collected from unvaccinated COVID-19 patients with confirmed Beta (*n* = 3) or Delta (*n* = 20) infection, and cross-neutralizing activity was determined using a matched selection of wave 1 (*n* = 20) and Alpha (*n* = 20) acute-phase serum samples collected 11–53 days post-symptom onset. Results showed that antibodies from infection with Alpha, Beta, or Delta cross-neutralized wild-type (WT) parental virus, but had reduced neutralization against divergent lineages. Antibodies from WT infection showed the greatest cross-neutralization of variant lineages, and infection-induced antibodies had greatly reduced cross-neutralization against Beta. These data suggest that vaccines based on the ancestral Wuhan strain are likely capable of inducing cross-reactive antibodies and protecting against variants (at least for Alpha, Beta, Gamma, Delta, and other pre-Omicron variants), compared with natural infection with a single variant. The second study examined cross-neutralization activity in convalescent sera pools grouped by exposure to ancestral (D614G; *n* = 10), Epsilon (B.1.429, *n* = 15), B.1.1.519 (*n* = 6), Zeta (P.2; *n* = 1), Iota (B.1.526; *n* = 1), and Delta (B.1.617.2; *n* = 3) variants against each distinct SARS-CoV-2-exposure variant, plus Beta, Gamma (P.1), B1.617, and Omicron [31]. Serum was also collected from eight patients with ancestral virus infection and subsequent BNT162b2 vaccination, and from 17 patients with Epsilon infection and subsequent BNT162b2 vaccination. In addition, serum was collected from 18 individuals who had no previous SARS-CoV-2 infection, 11 of whom had received two doses of BNT162b2, and seven of whom had received three doses of BNT162b2. The findings suggested that neutralizing-antibody responses were strongest against variants sharing the E484 spike-receptor-binding domain mutation (i.e., variants Delta, Gamma, Zeta, and Beta). Delta, Beta, and Omicron showed the greatest resistance to neutralization in serum from previously infected individuals, and previous infection exposure to the E484K mutation resulted in the greatest neutralization of variants with mutations at E484 (Delta, Gamma, Zeta, and Beta).

Similarly, two studies illustrated cross-reactive T-cell responses after infection [32,33]. In the first study, T-cell responses were evaluated in 68 unvaccinated hospitalized patients with COVID-19, post-infection with the ancestral, Beta, Delta, or Omicron SARS-CoV-2 variants, as well as 15 convalescent COVID-19 participants (ancestral strain, *n* = 7; Beta strain, *n* = 8 (based on the date of infection)) and 55 previously vaccinated patients (one dose of Ad26.COV2.S (*n* = 20; prior infection in 13), two doses of Ad26.COV2.S (*n* = 20; prior infection in 14), two doses of BNT162b2 (*n* = 15; prior infection in 6)) [32]. Omicron spike-specific CD4 T-cell responses in infected individuals were mostly targeted to conserved epitopes in spike proteins. Also, T-cell responses directed at spike, nucleocapsid and membrane proteins, in individuals infected during the Omicron-dominant fourth wave, were comparable with responses in patients infected with other variants. Interestingly, 70–80% of those T-cell responses (i.e., T-cell clones) were cross-reactive, despite Omicron’s ability to escape from neutralizing antibodies. The second study evaluated convalescent CD4+ and CD8+ T-cell responses to epitopes on spike only, and the entire SARS-CoV-2 proteome in 28 SARS-CoV-2-positive patients, 29 vaccinated individuals (~14 days after the second dose of an mRNA vaccine), and 23 unexposed individuals (samples taken May 2014–March 2018 or March–May 2020) [33]. SARS-CoV-2–specific memory T cells acquired through infection effectively recognized Alpha, Beta, Gamma, and California 20C clade (CAL.20C) variants, with 93–97% of CD4 and CD8 epitopes being conserved across variant proteomes. Similar T-cell reactivity and T-cell dose-dependent responses were observed against ancestral and variant spike peptides. There were also no differences in T-cell reactivity to the SARS-CoV-2 proteome between ancestral and variant strains, and a similar pattern of dominant antigen-induced T-cell activity was observed [33].

#### 2.2.3. Protection from Reinfection

As for the extent of protection from reinfection with new variants, varying degrees of variant escape from neutralization by antibodies in convalescent plasma from prior infection have been reported. The Beta variant appears to be largely resistant to convalescent sera from prior infection [34], and convalescent sera from unvaccinated individuals after Omicron infection did not generally cross-neutralize other variants [35]. This latter observation is supported by results from another study, which showed that Omicron BA.1 convalescent sera from unvaccinated individuals neutralized homologous BA.1 virus, but showed lower levels of neutralization of Omicron BA.2 and BA.3 variants; neutralizing geometric mean titers (GMTs) against heterologous BA.2, BA.3, and WT variants were 4.2-, 4.4-, and 28.4-fold lower, respectively, than those against homologous BA.1 [36].

Real-world studies provide evidence for protection of unvaccinated individuals against reinfection with new variants. In a pre-Omicron study of 25,661 healthcare workers (seropositive cohort, *n* = 8278; seronegative cohort, *n* = 17,383), seropositivity through infection robustly protected against asymptomatic and symptomatic reinfection [30]. Previous SARS-CoV-2 infection was associated with an 84% lower risk of infection, with the median protective effect occurring 7 months after primary infection (7.6 reinfections per 100,000 person–days in seropositive individuals versus 57.3 new infections per 100,000 person–days in seronegative individuals). Moreover, a 99.8% reduced risk in new infections was seen in seropositive individuals (probable reinfections). Similarly, a review of infection-induced immunity effectiveness and durability from studies in the pre-Omicron period, showed that previous infection provided 87.3% and 95.0% protection against asymptomatic and symptomatic reinfection, respectively, even more than one year after infection [37]. A national database study in Qatar showed that previous infection was 86–92% effective in preventing reinfection with Alpha, Beta, and Delta variants versus 56% effective against the Omicron variant [38]. Additionally, 87.8% effectiveness against severe, critical, or fatal disease caused by the Omicron variant was observed after previous infection.

A Nicaraguan study leveraged an established household influenza cohort (*n* = 2353, comprising newborn infants to the elderly up to 94 years, in 437 households) to compare the incidence of infection among seropositive (*n* = 1284) versus seronegative (*n* = 780) participants in March–November 2021, when Gamma and Delta variants were predominant [39]. Infection-induced immunity from the first wave in May 2020 provided an estimated 68.1% (95% CI: 59.2–75.1%) protection overall against symptomatic infection through to October 2021 (51.0% for ≤9 years, 73.3% for 10–49 years, 72.3% for ≥50 years), and substantially higher protection (overall, 78.9% (95% CI: 63.4–87.9%)) against moderate or severe infection. As described above, retrospective analyses of Qatar’s national databases have demonstrated pre-Omicron primary infection-derived effectiveness of ~85% against pre-Omicron reinfection and only ~38% against Omicron re-infection, both of which wane over time, but ~97% effectiveness against severe, critical, or fatal COVID-19 due to any variant reinfection, without evidence of waning [28]. Furthermore, in a separate analysis, pre-Omicron primary infection showed a low effectiveness of 35.5% (95% CI: 12.1–52.7%) against symptomatic reinfection with Omicron BA.4/BA.5 sub-variants, but an Omicron primary infection showed a much higher effectiveness of 76.2% (95% CI: 66.4–83.1%) against symptomatic BA.4/BA.5 reinfection [40].

Real-world evidence indicates that infection-acquired immunity to SARS-CoV-2 plays a role in reducing viral transmission. Among 1,789,728 individuals from 814,806 families (2–5 members) in Sweden (SARS-CoV-2 positive, *n* = 1,331,989; two-dose vaccinated, *n* = 3,640,421), during the Alpha predominant period, family members without immunity, who had at least one immune family member, had a 45–97% lower risk of contracting COVID-19 than those with no immune family members; a similar protective benefit was observed with immune family members from previous infection or vaccination [8]. Moreover, a significant inverse dose-response association was observed between the number of immune family members and the risk of a non-immune family member contracting COVID-19, irrespective of family size.

Although data among children are still limited, differences in adaptive immune response to SARS-CoV-2 between children and adults have been noted [41]. In children, both the antibody and cellular arms of the adaptive immune response against SARS-CoV-2 appear to be strong, sustained, and exhibit more cross-reactivity against other coronaviruses than in adults [41,42]. Spike-specific T-cell responses persisted >12 months in children, and were 2.1-fold higher in children (3–11 years) than in adults. Spike-specific T-cell responses observed in seronegative children suggest cross-reactive responses to seasonal coronaviruses.

Another study showed that antibodies produced by previous SARS-CoV-2 infection in children and adolescents did not neutralize Omicron, thereby rendering them susceptible to reinfection with Omicron [43]. Among 177 children and adolescents hospitalized due to acute COVID-19, MIS-C, or outpatients with mild convalescent COVID-19 (<5 years, *n* = 55; 5–11 years, *n* = 51; 12–21 years, *n* = 71), <10% of children showed neutralizing-antibody titers against the Omicron variant. In the acute COVID-19 cohort, children <5 years of age had lower neutralizing antibodies to viral variants than those aged 12–21 years. However, the study also showed that vaccination induced a much broader neutralizing-antibody response against variants of concern in naive adolescents than infection-derived immunity.

While it has been suggested that children may have protection against COVID-19 from pre-existing immunity to common cold coronaviruses [44], antibodies to seasonal human coronaviruses are rarely cross-reactive with SARS-CoV-2 [45]. Studies in adults have found that pre-existing humoral immunity to common coronaviruses did not provide cross-protection against SARS-CoV-2 [46], but pre-existing SARS-CoV-2 immunity did influence the potency, breadth, and durability of the humoral response to SARS-CoV-2 vaccination [47].

Real-world evidence on infection-induced immunity among children is available from a retrospective analysis of health records of ~300,000 unvaccinated uninfected versus previously infected children aged 5–18 years during the period of Delta predominance in Israel (July–December 2021) [48]. Overall, previous infection was ~80% effective against reinfection for ~18 months post-infection; effectiveness of previous infection-elicited immunity peaked at 89.2% (95% CI: 84.7–92.4%) at 3–6 months post-infection, waning to 82.5% (95% CI: 79.1–85.3%) at 9–12 months post-infection, and stabilizing for up to 18 months. Waning was seen more often among 12–18-year-olds and less frequently in 5–11-year-olds. From this analysis, it is unclear how the observed infection-elicited immunity compares with vaccination-elicited immunity, and immunity against the more immune-evasive Omicron variant.

### 2.3. Hybrid Immunity

#### 2.3.1. Immune Persistence and Quality

Understanding the durability of immunity derived from infection plus vaccination is possible by comparing the immune responses of three cohorts: unvaccinated with previous infection, vaccinated with previous infection, and vaccinated without previous infection. However, with the rapid rollout of COVID-19 mass-vaccination,, such studies are increasingly difficult to perform prospectively. One such study measured antibody persistence and the breadth of variant neutralization in a cohort of unvaccinated infected individuals (*n* = 62), of whom a subset (*n* = 28) subsequently received mRNA vaccination, between March and June 2020, before the emergence of SARS-CoV-2 variants of concern [49]. Previous infection plus two-dose mRNA vaccination demonstrated higher antibody titers that persisted for ~7 months post-symptom onset despite some waning, similar to responses from two-dose vaccination alone, and substantially higher than from infection alone. Over the same period, higher and sustained antibody-neutralizing activity across variants was observed in sera from previous infection plus vaccination, across Wuhan, D164G, Alpha, Beta, Gamma, and Delta pseudoviruses, though neutralization against Beta appeared the lowest. These observations indicate a priming role for infection-induced immunity and the booster effect of vaccination, and are supported by a clinical study demonstrating that vaccination of previously infected individuals conferred additional protection [50].

A systems-serology approach was used to elucidate the influence of prior SARS-CoV-2 infection on the vaccination-induced breadth of humoral and cellular immune effector responses [50]. Analysis of 68 mRNA vaccinees (*n* = 49 never infected and *n* = 14 infected prior to vaccination) found qualitatively superior antibody responses in individuals with hybrid immunity. Specifically, these responses corresponded to enhanced engagement of FcγR2a and FcγR3a antibodies that: may facilitate viral clearance by killing infected cells; and an improved ability to bind the S2 epitopes on the spike protein that is more conserved across the variants, but likely not accessible on most COVID-19 vaccine antigens bearing the pre-fusion-stabilized spike protein where S1 is immunodominant. Both immune responses could be advantageous to overcome immune-evasive variants.

#### 2.3.2. Breadth of Coverage of Variants

Three studies showed that combined immunity from infection plus vaccination demonstrates broad neutralization of homologous and heterologous variants [51,52,53]. SARS-CoV-2 infection before or after vaccination significantly boosted neutralizing-antibody responses, eliciting higher GMTs in individuals with hybrid immunity of 10.8-fold for WA1, 16.9-fold for Alpha, 32.8-fold for Beta, and 15.7-fold for Delta variants versus vaccination alone, and higher GMTs in individuals who experienced postvaccination infection of >6.0-, 11.8-, 17.0-, and 8.5-fold versus vaccination alone, respectively [51]. Serum antibody potency was greater for hybrid immunity and postvaccination infection groups, and showed variant-neutralizing titers. In another study, Beta infection plus BNT162b2 mRNA vaccination, specifically, was associated with a >3-fold reduction in Omicron escape, compared with Beta infection alone [53]. Omicron-neutralizing GMTs increased 69.3-fold postvaccination in Beta-infected individuals, and Beta infection alone showed decreased cross-neutralization activity against Omicron compared with Beta infection plus vaccination [53]. The third study showed that mRNA vaccination plus SARS-CoV-2 infection (Alpha, Beta, Gamma, Delta, or Omicron variants) led to broad neutralizing capacity, with robust neutralization demonstrated for heterologous variants [52]. Infection with the Delta variant after two-dose mRNA vaccination resulted in a 12.5-fold reduction in neutralization of Omicron, while post-vaccination infection with Omicron resulted in a smaller 1.5-fold reduction in neutralization of Delta. Additionally, the fold reduction in neutralizing activity was higher in postvaccination sera than in convalescent (vaccination plus infection) sera. Previous SARS-CoV-2 infection plus vaccination with BNT162b2 mRNA vaccine was associated with increased memory T-cell response to Omicron; polyfunctional vaccine-induced memory T cells responded to a variety of SARS-CoV-2 variants, even if neutralizing antibodies could not neutralize them [54].

Several studies have evaluated immunity elicited by infection plus a single dose of vaccine, and found that single-dose hybrid immunity enhances infection-elicited immune response [55,56]. After one dose of BNT162b2, individuals with prior SARS-CoV-2 infection showed enhanced T-cell immunity, antibody-secreting memory B-cell response to the spike protein, and neutralizing antibodies effective against Alpha and Beta variants [56]. Additionally, single-dose vaccination with BNT162b2 in the context of prior infection with a heterologous variant substantially enhanced neutralizing-antibody responses against variants, and there was no additional improvement after the second vaccine dose [55]. Similarly, although the IgG concentrations in previously infected subjects after one or two doses of BNT162b2 mRNA vaccine were similar, one-dose BNT162b2 vaccinees with previous SARS-CoV-2 infection elicited 3-fold higher neutralizing antibodies compared with uninfected two-dose vaccinees [57]. Neutralizing-antibody titers against the ancestral virus due to hybrid immunity were higher after severe versus mild COVID-19 experience, with cross-neutralizing-antibody titers also higher against Alpha, Beta, and Delta variants but markedly lower against Omicron BA.1. Interestingly, SARS-CoV-1 survivors immunized with only a single-dose of BNT162b2 mRNA vaccine showed induction of potent pan-sarbecovirus neutralizing antibodies, suggesting a cross-clade booster effect [58]. Furthermore, B cells recognizing spike-receptor-binding domains, from both SARS-CoV-1 and SARS-CoV-2, were significantly enriched among individuals with previous SARS-CoV-1 infection plus BNT162b2 vaccination versus BNT162b2 vaccinees with previous SARS-CoV-2 infection and BNT162b2 vaccinees without previous SARS-CoV-2 infection; previous infection plus two-dose BNT162b2 vaccination elicited enhanced levels of neutralizing antibodies against the SARS-CoV-2 clade [58]. Finally, a longitudinal study evaluated infection-induced and hybrid immunity during four COVID-19 waves in a South African cohort, and observed a progressive increase in seropositive-status [59]. Spike antibodies elicited during a prior wave were protective during the subsequent infectious wave for all variants except Omicron. After one dose of BNT162b2 vaccine, anti-spike IgG responses were significantly higher in seropositive versus seronegative individuals and above the threshold of 50% protection against Omicron infection; however, a second vaccine dose in seropositive individuals did not significantly increase anti-spike IgG levels.

#### 2.3.3. Effect on Reinfection

Lessons from the first outbreak caused by the Delta variant in a highly vaccinated population in Provincetown, MA, USA, indicated that infections in previously vaccinated individuals triggered robust anamnestic immune responses, including both B-cell and T-cell responses against ancestral and Delta variants [60]. Of 469 cases, 346 (74%) were fully vaccinated; 274 of 346 (79%) vaccinated patients with breakthrough infections were symptomatic, but only 4 (1.2%) were hospitalized, demonstrating the benefit of vaccination on post-vaccination SARS-CoV-2 infections [61]. A second study showed that previous exposure to SARS-CoV-2, followed by a spike-based protein vaccine (SCB-2019), decreased the risk and severity of subsequent COVID-19 infection [62]. Data from 14,757 participants demonstrated that previous exposure to infection provided an estimated protection of 83.2% against COVID-19 of any severity and 100% protection against severe COVID-19 illness or COVID-19-related hospitalization; enhanced protection was further observed with one dose (49.9%) and two doses (64.2%) of the SCB-2019 vaccine.

A study conducted using Bahrain’s national health databases examined reinfection frequency from April 2020 through to July 2021 among individuals of varied vaccination statuses (fully vaccinated, interrupted vaccination, one-dose vaccination, post-reinfection vaccination, and unvaccinated); this study found that both immunity through vaccination and prior infection were effective in reducing the severity of reinfection [63]. A total of 6.6% of the reported reinfection cases required hospitalization, and the 1 death occurred in the unvaccinated group. The reinfection cases were mild in severity, and vaccinated individuals were less likely to show symptoms. Similarly, in Sweden, one-dose and two-dose hybrid immunity was associated with a 58% and 66% reduced risk of reinfection compared with infection-induced immunity, respectively [29]. A risk reduction after one dose was observed for up to two months, with evidence of attenuation for up to nine months, whereas two-dose immunity showed no significant attenuation for up to nine months. Additionally, hybrid immunity resulted in a lower risk of COVID-19-associated hospitalization compared with infection-induced immunity, regardless of the number of doses. Furthermore, a study from Rhode Island, USA, evaluating population-based estimates of SARS-CoV-2 reinfection and effectiveness of vaccination after COVID-19 infection, demonstrated a relatively high risk of reinfection among unvaccinated individuals, and an ~50% lower risk of reinfection in individuals vaccinated after recovery from COVID-19 [64].

#### 2.3.4. Effect on Transmission

Real-world evidence concerning the effects of hybrid immunity on blocking transmission is available from a Belgian contact-tracing study, conducted between 26 January 2021 and 14 December 2021 (pre-Omicron period), which demonstrated that significant protection remained following vaccination despite reduced protection associated with infection with the Delta variant and time since vaccination [65]. Hybrid immunity offered more protection against transmission of SARS-CoV-2 variants than previous infection or vaccination alone, where VE after hybrid immunity persisted to a lower extent 0–50 days and 150–200 days after infection or vaccination. Further real-world evidence concerning the effects on transmission showed a BNT162b2 average VE of ~80% against household infection, i.e., transmission from infected household contacts, when comparing fully vaccinated individuals to those who were unvaccinated (80.3%) or vaccinated once (82.0%) within 7 days of the original infection [66]. The relatively high VE rates demonstrate the high degree of protection achieved by vaccination even against high-risk exposure, that in turn helps reduce viral circulation.

#### 2.3.5. Omicron and Hybrid Immunity

Hybrid immunity results in strong neutralizing activity against Omicron BA.1 and BA.2 in the plasma, and post-vaccination Omicron infections induce neutralizing activity in the nasal mucosa, which is not observed with vaccination alone [67]. Further, primary infections with Omicron may only elicit narrow specificity of memory B cells. Accordingly, a study showed that postvaccination infection with Omicron BA.1 mainly recalls humoral immune memory against WT spike protein [68]. Vaccination plus Omicron BA.1 infection enhanced BA.1 neutralization and cross-neutralization of D614G and Omicron BA.2.

Post-vaccination Omicron BA.2 infection resulted in broad neutralizing activity and enhanced cross-neutralization of BA.2.12.1 and BA.4/BA.5 sub-variants [69]. Post-vaccination infection with BA.2 was shown to be more efficient in cross-neutralization than infections with the BA.1 variant, most probably due to BA.1 infections not eliciting a stronger recall of B cells. Moreover, Omicron sub-lineages BA.4/BA.5 appear to escape BA.1 infection-elicited neutralizing antibodies in unvaccinated individuals [70], suggesting that BA.1 infection alone does not protect against other Omicron BA lineages. Another study evaluating three-dose mRNA vaccinee serum found that sub-lineages BA.4/BA.5 resisted neutralization more than BA.1 and BA.2, and the sera from post-vaccination BA.1 infections showed reduced neutralization of BA.4/BA.5 when compared with BA.1 and BA.2 [71].

Additional booster doses of the COVID-19 vaccine based on the ancestral SARS-CoV-2 strain have been able to restore waning immunity, including against some sub-lineages of the antigenically divergent Omicron variant. Individuals vaccinated with two or three doses of BNT162b2 exhibited strong antibody and memory B-cell responses in response to post-vaccination infection with Omicron BA.1, and protection against Omicron infection was greatly boosted with a third dose of mRNA vaccine [72]. Interestingly, post-vaccination infection by Omicron BA.1 elicited robust neutralizing titers against Omicron sub-lineages BA.1, BA.2, and previous variants of concern, but not against BA.4/BA.5 variants. Furthermore, three-dose BNT162b2 vaccinees with or without BA.1/BA.2 post-vaccination infection showed a reduced neutralization of Omicron BA.4/BA.5 versus other Omicron sub-lineages [73].

Stronger neutralization evasion has been reported for Omicron BA.2.12.1 and BA.4/BA.5 when compared with BA.2 neutralization by the three-dose vaccinee sera, and more interestingly, from BA.1 infections post-vaccination [68]. Moreover, BA.1 infection elicited BA.1-specific antibodies with a narrow breadth of coverage that strongly neutralized BA.1, but not BA.2 and BA.4/BA.5, and may have been weak against pre-Omicron variants [68]. A study evaluating neutralization activity against Omicron sub-lineages and the “Deltacron” SARS-CoV-2 variant from individuals vaccinated with three doses of BNT162b2, or unvaccinated with BA.1 infection, reported that the sera from BA.1 infection did not have robust neutralization against Omicron BA.4/BA.5 variants [74].

Real-world studies provide insights into both the evolution of vaccine-mediated protective immunity over time, and the protective effects of previous infection on reinfection that may be important considerations when designing new/updated vaccines. A study evaluating the effectiveness of previous infection, BNT162b2 vaccination, and hybrid immunity against Omicron infection and severe, critical, or fatal COVID-19 in Qatar showed that VE after two doses of BNT162b2 in individuals with no previous infection was negligible (−1.1%; 95% CI: −7.1% to 4.6%), but almost everyone had received their second dose >6 months earlier [75]. After three doses of BNT162b2, VE increased to 52.2% (95% CI: 48.1–55.9%) in individuals with no previous infection. However, in previously infected individuals, two-dose VE was 55.1% (95% CI: 50.9–58.9%), and three-dose VE was 77.3% (95% CI: 72.4–81.4%) [75]. Another real-world study by the same group estimated that previous infection with SARS-CoV-2 was roughly 90% effective in preventing a reinfection by pre-Omicron variants in both vaccinated and unvaccinated people; however, after Omicron emerged, prior infection only provided ~60% protection against reinfection [38]. Real-world evidence also suggests that reinfection can occur as soon as 3 months after contracting COVID-19 [76]. Indeed, in a study from Denmark, some people were reinfected with the BA.2 sub-lineage of Omicron as few as 20 days after infection with Omicron BA.1 [77].

Individuals vaccinated with two or three doses of BNT162b2 experience strong antibody and memory B-cell responses against the Omicron variant after a post-vaccination BA.1 infection, and protection against Omicron infection is greatly boosted with a third-dose vaccination; however, this immunity wanes over time [72,78,79].

### 2.4. New COVID-19 Vaccines

#### 2.4.1. Design Considerations for New COVID-19 Vaccines

Accumulating data on immunity derived from infection or vaccination only, and from hybrid immunity, helps inform the design considerations for the next generation of vaccines to overcome the rapid evolution exhibited by SARS-CoV-2 and confer immunity to current immune evasive variants and ideally future variants. Two investigations into the SARS-CoV-2 antigenic landscape provide some insights. The first study showed that unvaccinated individuals who suffered Omicron BA.2 infection induced a low neutralization of both pre-Omicron and Omicron BA.1 variants, and that cross-neutralizing antibody levels across the entire antigenic space were greatly increased after ≥3 antigenically close variant exposures (vaccination or infection) or after 2 exposures (vaccination or infection) to antigenically distinct variants [80]. The second study also used antigenic cartography to evaluate neutralizing antibodies after 1 or multiple exposures [81]: three-dose mRNA vaccination resulted in a lower breadth of neutralization across Omicron sub-lineages BA.4/BA.5 and BA.2.12.1 when compared with the BA.1 variant; also BA.1 infection post-three-dose vaccination elicited broader immunity against the whole antigenic space when compared with three-dose vaccination alone.

Evidence to date suggests that in naive individuals, Omicron-only vaccines are unlikely to be superior to the current vaccines based on the ancestral SARS-CoV-2 strain. Omicron infection in unvaccinated persons triggers antibody responses that show neutralization limited to the Omicron variant only and poor cross-reactivity against other variants of concern [82,83]. This lack of cross-neutralization was confirmed in another study that showed that an Omicron infection alone did not enhance antibody response to the other variants (WT, Delta, Omicron) [84]. In contrast, post-vaccination Omicron BA.1 infection [82], and post-vaccination Delta or Omicron infection, induced higher and broad neutralization against variants of concern versus in unvaccinated individuals [83].

Bivalent vaccines, on the other hand, can provide a breadth of immunity by eliciting immune responses against the ancestral strain antigens, as well as Omicron-specific divergent epitopes. In contrast with current monovalent vaccines, which are based on the spike protein of the Wuhan ancestral strain, the term “bivalent” means that the vaccine is derived from two strains of SARS-CoV-2: the original variant (Wuhan or WT), and the epidemiologically prevalent Omicron strain, i.e., BA.4 and BA.5 subvariants, which became predominant mid-2022.

Omicron-containing mRNA COVID-19 vaccines have been described in clinical studies. Given as a fourth dose in participants >55 years of age without evidence of prior infection, a 30-µg bivalent vaccine (BNT162b2 15 µg + Omicron BA.1 15 µg) was shown to meet superiority criteria versus a 30-µg monovalent BNT162b2 vaccine in terms of the geometric mean ratio (GMR; 95% CI lower bound >1.0) of elicited neutralizing antibodies against Omicron BA.1, with a GMR of 1.56 (95% CI: 1.17–2.08); a 60-µg bivalent dose also met superiority criterion with a GMR of 1.97 (95% CI: 1.45–2.68) [85]; geometric mean fold rises (GMFRs) in neutralizing activity pre-vs post-vaccination were 9.1-fold (30-µg bivalent dose) and 10.9-fold (60-µg bivalent dose). The Omicron BA.1-containing bivalent vaccine elicited lower neutralization responses against BA.4/BA.5 variants compared with the BA.1 variant, regardless of the vaccine dose level. Consistent with the bivalent BNT162b2 study, preclinical data in mice, using an Omicron BA.4/BA.5 component plus the original vaccine component, seem to provide a high level of neutralizing-antibody titers against BA.4/BA.5 sub-lineages and a broad response against other Omicron sub-lineages, as well as a robust response against the original strain [86].

Similar results were obtained with a fourth dose of mRNA-1273.214 Omicron-containing mRNA COVID-19 vaccine at 50-µg dose levels (25 µg ancestral mRNA-1273 + 25 µg Omicron BA.1), i.e., a higher Omicron BA.1 neutralizing-antibody titer versus original mRNA-1273 in participants with and without prior infection [87]. Administration of the original booster (fourth dose) elicited a 3.8-fold rise in GMTs, whereas the bivalent Omicron BA.1-containing vaccine booster showed a 7.1-fold rise. The mRNA-1273.214 bivalent vaccine, administered as a fourth dose, also elicited higher binding-antibody titers across previous variants of concern (i.e., Alpha, Beta, Delta, and Gamma). The mRNA-1273.214 booster further enhanced the neutralizing titers against Omicron BA.1 while retaining a noninferior titer against the ancestral virus [88]. In addition, this bivalent BA.1-based vaccine boosted neutralizing titers against BA.4/BA.5 by 5.4-fold (95% CI: 5.0–5.9) above baseline in all participants regardless of prior infection, and by 6.3-fold (95% CI: 5.7–6.9) in seronegative participants.

Beyond BA.4/BA.5, a number of new Omicron subvariants are circulating that exhibit increasing antigenic divergence from the ancestral strain and previous variants. Studies have evaluated the benefit provided by bivalent mRNA vaccines against these new Omicron sub-lineages in terms of antibody neutralization activity. Zou et al. (2022) compared sera from adult vaccinees >55 years old who had received three doses of the monovalent BNT162b2 mRNA vaccine plus a fourth dose of the monovalent BNT162b2 mRNA vaccine, versus a fourth dose of a BA.4/BA.5-containing bivalent vaccine [89]. The bivalent vaccine elicited higher neutralizing titers against new emerging Omicron sub-lineages, BA.4/5, BA.4.6, BA.2.75.2, BQ.1.1, and XBB.1 (GMFR in titers for the bivalent vaccine: 13.0x, 11.1x, 6.7x, 8.7x and 4.8x versus the monovalent vaccine: 2.9x, 2.3x, 2.1x, 1.8x, and 1.5x, respectively), regardless of SARS-CoV-2 infection history. Davis-Gardner et al. (2022) used sera from three cohorts of two-dose vaccinees who had received: a third dose monovalent booster (*n* = 12), third and fourth dose monovalent boosters (*n* = 11), and a third dose bivalent booster, to examine in vitro neutralization of live, patient-derived viruses of the Omicron sub-lineages BA.1, BA.5, BA.2.75.2, BQ.1.1, and XBB [90]. Although the one-dose bivalent booster elicited higher neutralizing titers compared with the one- or two-dose monovalent booster against all the Omicron subvariants, overall neutralizing titers were much lower than against the ancestral strain (23 to 63 times lower for BA.2.75.2, BQ.1.1, and XBB).

Early data on the real-world effectiveness of BA.4/5-containing bivalent booster vaccines is also available from the US. Among adults ≥65 years, VE against hospitalization, measured during 8 September to 30 November 2022, was 84% (95% CI: 64–93) compared with no prior vaccination and 73% (95% CI: 52–85) compared with at least two-dose monovalent mRNA vaccination [91]. Among adults ≥18 years, VE against emergency room or urgent care visits, measured between 13 September and 18 November 2022, was 56% (95% CI: 49–62) compared with no prior vaccination, and 31–50% (95% CI: 19–57) compared with two-dose monovalent mRNA vaccination [92].

#### 2.4.2. Regulatory Considerations for New COVID-19 Vaccines

Evolving positions and advice of regulators and global vaccine technical committees are critical to determine necessary changes to the current vaccination programs that may help in continued protection against current and emerging COVID-19 variants.

As of December 2022, bivalent Omicron BA.4/BA.5 vaccines are authorized for use as a booster dose in all individuals >5 years of age by the US FDA [93] and EMA [94], following the specific age-appropriate dose, to protect against COVID-19 caused by the Omicron variant. Similarly, the World Health Organization Technical Advisory Group on COVID-19 Vaccine Composition (WHO-TAG-CO-VAC) has indicated that vaccines with a WHO Emergency Use Listing (EUL), or any of the authorized bivalent variant-containing vaccines, can be used as boosters to protect against severe disease caused by variants of concern; specifically, a bivalent booster may be offered to all individuals >12 years of age, prioritizing high risk sub-populations [95].

#### 2.4.3. Implementation Considerations for New COVID-19 Vaccines

Implementation, priority groups, and target age groups could vary across countries. For example, recommendations from the Joint Committee on Vaccination and Immunisation (JCVI) in the UK for their booster program include the use of either the Pfizer-BioNTech BNT162b2 monovalent mRNA vaccine, the Moderna mRNA-1273 original WT monovalent, or the mRNA-1273 bivalent (WT + Omicron BA.1) vaccine in adults ≥18 years [96]. For those aged 5–11 and 12–17 years, JCVI advises the use of corresponding Pfizer-BioNTech BNT162b2 monovalent WT mRNA vaccine. The US CDC and Advisory Committee on Immunization Practices updated recommendations for booster vaccination (as of 19 October 2022) state that only bivalent mRNA COVID-19 vaccines should be used as a booster dose for all individuals ≥5 years of age after completion of a monovalent primary series or receipt of monovalent booster dose(s); in addition, monovalent vaccines based on the original (ancestral) strain of SARS-CoV-2 are no longer recommended as a booster dose for those 5 years and older [97,98]. More recently, the EMA Emergency Task Force (ETF) considered that the bivalent original/Omicron BA.4/5 vaccines may be used in previously unvaccinated children and adults to provide broad protection [99].

#### 2.4.4. Evolution of Hybrid Immunity with New COVID-19 Vaccines

Finally, an important consideration when designing new COVID-19 vaccines is how vaccine-mediated protective immunity will evolve over time and be modified by iterations of exposure to COVID-19 vaccines and infection with increasingly divergent viral variants. Post-vaccination infections appear to induce serum-binding and serum-neutralizing antibody responses that are markedly more potent, durable, and resilient to variant spike mutations than those observed in two-dose vaccinees [100]. However, individuals with post-vaccination infection, those who were vaccinated after infection, and three-dose vaccinees show serum-neutralizing activity of comparable magnitude and breadth, suggesting that an increased number of exposures to SARS-CoV-2 antigens can enhance the quality of antibody responses [100]. A longitudinal study in healthy mothers investigated the effects of infection, illness, and serological responses on natural exposure to SARS-CoV-2 over four successive waves of variants in South Africa, and the impact of prior natural exposure on BNT162b2 mRNA vaccine responses [59]. Seropositivity induced by natural exposure to the SARS-CoV-2 virus conferred protection from subsequent infection with Beta and Delta variants but not Omicron, for which only very high levels of natural antibody provided protection. Vaccination of seropositive individuals elicited higher concentrations of spike IgG than vaccination of seronegative individuals, and a greater proportion of seropositive, vaccinated mothers were protected from Omicron, reiterating the observation that a single dose of ancestral SARS-CoV-2 strain-based vaccine may contribute to protection against known or related SARS-CoV-2 variants in seropositive individuals.

Similar evidence is available from three carefully designed real-world studies. A nationwide test-negative case-control study using the federated health databases of Qatar showed that previous SARS-CoV-2 infection can help prevent reinfection with Omicron subvariants BA.4 and BA.5; effectiveness of prior infection with a pre-Omicron variant was 35.5% (95% CI: 12.1–52.7) against symptomatic BA.4 or BA.5 reinfection, and 27.7% (95% CI: 19.3–35.2) regardless of the presence of symptoms [40]. However, a post-Omicron (including BA.1 or BA.2) variant infection showed higher effectiveness against BA.4/5 reinfection: 76.2% (95% CI: 66.4–83.1) against symptomatic reinfection and 78.0% (95% CI: 75.0–80.7), regardless of the presence of symptoms. Furthermore, this protection waned over time since prior infection, supporting the inference that both waning of infection-induced immunity and the antigenic divergence of emerging variants, such as Omicron, influence the level of protection from reinfection. A retrospective cohort design study analyzing a captive population (~60,000 residents) in a congregate setting (i.e., the California prison system) between 24 December 2021, and 14 April 2022, showed that previous SARS-CoV-2 infection was effective in preventing reinfection with Omicron, and prior infection plus vaccination was even more effective [101]. Prior infection without vaccination showed effectiveness against Omicron reinfection of 27.5% (95% CI: 14.8–38.4) for residents infected before the Delta era, and 38.3% (95% CI: 6.5–59.3) for those with likely Delta infections—illustrating again the influence of both waning of infection-induced immunity and antigenic divergence of the Omicron variant. Effectiveness of mRNA vaccination without evidence of prior infection was 18.6% (95% CI: 7.7–28.1) with two doses, and increased to 40.9% (95% CI: 31.9–48.7) with three doses. Moreover, effectiveness of mRNA vaccination plus prior Delta infection was much higher: 68.7% (95% CI: 38.5–84.1) with two doses, and rising even higher to 84.6% (95% CI: 70.7–91.9) with three doses. Another study within the California prison system conducted between December 2021 to May 2022, during the period of Omicron predominance, showed that hybrid immunity demonstrates substantial effectiveness against transmission of SARS-CoV-2. Prior infection plus vaccination was 40% (95% CI: 20–55) effective in reducing the risk of transmitting Omicron infection, while vaccination alone was 22% (95% CI: 6–36) effective, and prior infection alone was 23% (95% CI: 3–39) effective [102]. Thus, hybrid immunity derived from combining infection and vaccination achieved high levels of protection against reinfection with antigenically divergent variants like Delta and Omicron.

## 3. Perspectives

**Figure 2:**
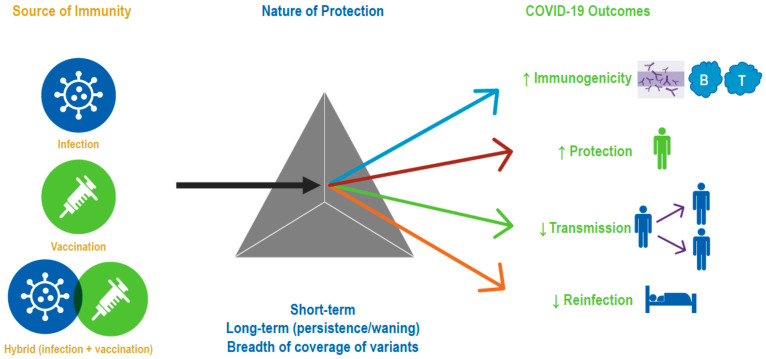
Evidence synthesis approach/strategy. The gray triangle symbolizes three types of immune protection, namely that obtained through vaccination, infection, or a combination of both. Accumulating data have enhanced our understanding of the Nature of Protection conferred by the three types of immunity against various COVID-19 outcomes.

**Figure 3:**
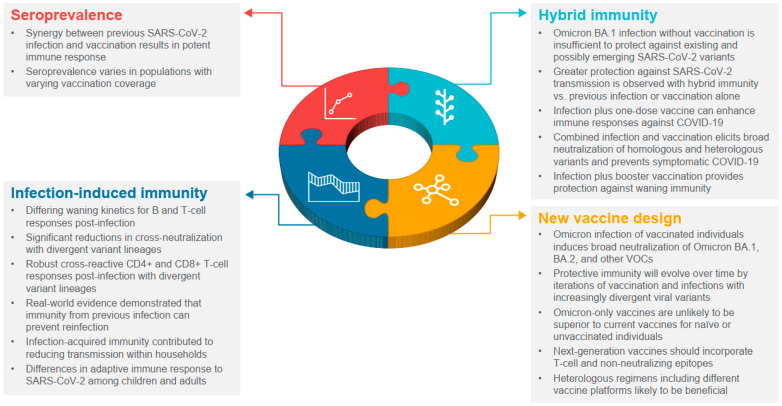
Lessons learned from widespread infection and vaccination. VOC, variants of concern.

In a little over 2 years, the global population has gone from immunologically naïve to SARS-CoV-2, to acquiring varying degrees of immunity from infection and/or vaccination. Increasingly, the global population is acquiring hybrid immunity from both booster vaccinations and infection with variants, such as Omicron, that are transmissible and immune evasive, with waning immunity post-primary vaccination. In this narrative review, we sought to summarize our growing understanding of SARS-CoV-2 infection-induced and hybrid immunity in terms of data on several immunogenicity—both B- and T-cell responses, immune persistence and memory responses, and the breadth of protection across variants of concern. The key findings are summarized in Figure 2 and Figure 3.

Prospective and real-world studies demonstrated durable protection against symptomatic disease following previous SARS-CoV-2 infection and booster vaccination [101,103,104], with vaccination of previously infected individuals providing protection for 6–8 months [103,104]. mRNA boosters provided additional protection against Omicron, including among individuals with prior infection [101]. Hybrid immunity with boosters also provides protection against waning immunity [105,106,107]. Vaccination after natural infection protected against severe disease [105,106], and the risk of reinfection and hospitalization was decreased with hybrid immunity [29,107].

Improved versions of vaccines include variant-specific (e.g., Omicron) and bivalent (e.g., ancestral plus Omicron variant) versions; that said, additional enhanced or next-generation COVID-19 vaccines include, for example, incorporating T-cell epitopes (i.e., non-neutralizing epitopes conserved in the nucleocapsid protein that are important for immune effector signaling [3]). In determining the role of new vaccines in helping to provide robust immunity to SARS-CoV-2 going forward, it is essential to utilize the power of hybrid immunity by intelligently augmenting widespread infection-elicited immunity with a rational use of existing or new vaccines [108]. Intranasal vaccines aimed at eliciting mucosal immunity to reduce or prevent SARS-CoV-2 infection appear promising, but initial results in human clinical trials have been mixed [109,110]. Optimizing heterologous regimens utilizing different vaccine platforms could help enhance the breadth of vaccine-induced immunity. Additionally, the use of novel vaccines with antigens from multiple variants, development of a pan-SARS-CoV-2/pan-sarbecovirus vaccine, or the implementation of novel delivery mechanisms, may help to interrupt transmission [108].

## 4. Conclusions

With the progression of the pandemic, several lessons have been learned with respect to infection-induced immunity. Specifically, there has been an improved understanding of the duration/kinetics of antibody decay [26] and the breadth of immune coverage against SARS-CoV-2 variants [30,31,32,33]. Studies have described both the differences and similarities in the adaptive immune responses to infection between children and adults [38,41]. In addition, real-world studies have provided valuable information about rates of reinfection with new variants, the effectiveness of infection-induced immunity for the prevention of subsequent symptomatic COVID-19 [30,38,40], and its role in reducing SARS-CoV-2 transmission [8,65].

For natural immunity, immunologic memory has been observed for >8 months for CD4+ T cells, CD8+ T cells, memory B cells, and antibodies [111]. Protection against symptomatic infection is 93–100% over 7–8 months, but there is evidence of more reinfections with Beta, Gamma, Iota, and Delta variants.

With respect to vaccine-induced immunity, most of the available COVID-19 vaccines consist of a single antigen, restricting epitope breadth of CD4+ and CD8+ T-cell responses compared with natural infection. Vaccine regimens split into two or three immunizations have been successful due to the heightened response after repeated exposure, and heterologous prime-boost regimens can result in stronger immune responses.

Synergy between previous SARS-CoV-2 infection and vaccination results in “hybrid vigor immunity,” which elicits a potent immune response. An increase in variant-neutralizing antibodies after vaccination of previously infected individuals recalls diverse and high-quality memory B cells generated after initial infection. Also, memory B cells are increased 5- to 10-fold in hybrid immunity when compared with natural immunity or vaccination alone, and enhanced vaccine responses have been observed after both asymptomatic and symptomatic SARS-CoV-2 infections.

## Data Availability

Not applicable.
